# Confiabilidade e Concordância Intra-Avaliador das Avaliações da Pressão Arterial, Rigidez Arterial e Variabilidade da Frequência Cardíaca em Pacientes com Doença de Parkinson

**DOI:** 10.36660/abc.20240132

**Published:** 2024-10-23

**Authors:** Verônica de Fátima Souza Lima, Rafael Yokoyama Fecchio, Maria Elisa Pimentel Piemonte, Marilia de Almeida Correia, Hélcio Kanegusuku, Raphael Mendes Ritti-Dias

**Affiliations:** 1 Universidade Nove de Julho São Paulo SP Brasil Universidade Nove de Julho, São Paulo, SP – Brasil; 2 Universidade de São Paulo São Paulo SP Brasil Universidade de São Paulo, São Paulo, SP – Brasil; 3 Hospital Israelita Albert Einstein São Paulo SP Brasil Hospital Israelita Albert Einstein, São Paulo, SP – Brasil

**Keywords:** Doença de Parkinson, Pressão Arterial, Rigidez Vascular, Frequência cardíaca

## Abstract

Avaliar a confiabilidade e a concordância intra-avaliador das avaliações da pressão arterial (PA), rigidez arterial e variabilidade da frequência cardíaca (VFC) em pacientes com doença de Parkinson (DP). Vinte pacientes com DP realizaram três visitas ao laboratório, durante as quais foram realizadas avaliações da PA braquial e central (tonometria de aplanação e auscultatório, respectivamente), rigidez arterial (velocidade da onda de pulso carotídeo-femoral e índice de aumento) e VFC em repouso. A PA sistólica braquial e central apresentou valores maiores na visita 1 quando comparada às visitas 2 e 3 (122±13 vs. 116±16 vs. 120±15, p=0,029). Não houve diferenças significativas (p>0,05) entre as visitas experimentais para outros parâmetros. A PA braquial e central apresentou um coeficiente de correlação intraclasse (CCI) acima de 0,842 e um erro padrão de medida (EPM) menor que 5,0%. Os gráficos de Bland–Altman indicaram baixa concordância entre as visitas 1 e 2 e boa concordância entre as visitas 2 e 3. Os índices de rigidez arterial exibiram valores de CCI entre 0,781 e 0,886, e o EPM variou de 7,3% a 25,2%. Os gráficos de Bland–Altman indicaram concordância moderada a boa entre as visitas para os parâmetros de rigidez arterial. Os índices de VFC apresentaram valores de CCI variando de 0,558 a 0,854 e valores de EPM que variam de 5,1% a 76,0%. Os gráficos de Bland–Altman indicaram concordância moderada entre as visitas para os parâmetros de VFC. Em pacientes com DP, a PA braquial e central apresenta baixa confiabilidade intra-avaliador e concordância entre as visitas 1 e 2 e boa confiabilidade intra-avaliador e concordância entre as visitas 2 e 3. Em geral, as avaliações de rigidez arterial e VFC apresentam confiabilidade intra-avaliador e concordância aceitáveis entre as visitas, exceto para o equilíbrio simpático-vagal cardíaco.

## Introdução

A doença de Parkinson (DP) é um distúrbio neurodegenerativo progressivo, caracterizado pela disfunção do sistema dopaminérgico nigroestriatal, que resulta em sintomas motores como bradicinesia, tremor de repouso, rigidez e instabilidade postural,^1^ reduzindo a qualidade de vida desses pacientes.^2^ A DP também envolve a degeneração do sistema nervoso autônomo periférico, incluindo reduções das fibras noradrenérgicas e da disponibilidade de norepinefrina no miocárdio, o que contribui para a desregulação cardiovascular.^1^ Evidências emergentes mostram que alterações na função e regulação cardiovascular podem estar associadas aos sintomas debilitantes da DP.

Em pacientes com DP, a pressão arterial (PA) elevada em repouso aumenta o risco de comprometimento cognitivo leve em quatro vezes.^3^ O aumento da rigidez arterial está associado à hipotensão ortostática e hipertensão supina,^4^ enquanto a modulação autonômica cardíaca reduzida está ligada ao congelamento da marcha^5^ e ao comprometimento cognitivo.^6^ Consequentemente, existe um interesse crescente em avaliar os resultados cardiovasculares em pacientes com DP. Apesar disso, não está claro se as avaliações de variáveis cardiovasculares apresentam boa confiabilidade e concordância intra-avaliador, que são fatores essenciais para avaliar as reais mudanças em resposta às intervenções. Sendo assim, no estudo atual, avaliamos a confiabilidade e a concordância intra-avaliador da PA braquial e central (tonometria de aplanação e auscultatório, respectivamente), rigidez arterial, velocidade da onda de pulso carotídeo-femoral e índice de aumento e avaliações da variabilidade da frequência cardíaca (VFC) em pacientes com DP.

## Métodos

### Participantes

O presente estudo é uma análise secundária de dados obtidos a partir de um estudo publicado anteriormente.^7^ Para esse estudo, o poder obtido foi de 0,99%, com um tamanho de efeito de 0,70 para a resposta da PA sistólica braquial para as três sessões experimentais realizadas por 20 pacientes, considerando um erro alfa de 0,05 (G*Power v. 3.1.9.4, Universität Kiel, Alemanha). Assim, o tamanho da amostra do presente estudo foi o mesmo do anterior. Uma amostragem não probabilística foi usada para recrutar pacientes da Associação Brasileira de Parkinson em São Paulo, Brasil. Os critérios de elegibilidade incluíram o diagnóstico confirmado de DP, idade mínima de 50 anos, não estar nos estágios 4-5 da DP (de acordo com a escala modificada de Hoehn e Yahr), não ter qualquer outro distúrbio neurológico além da DP, não ter qualquer doença cardíaca ou anormalidades eletrocardiográficas em repouso e durante testes de exercício máximo e não usar medicamentos que impactam diretamente a regulação autonômica cardíaca (por exemplo, betabloqueadores), exceto aqueles prescritos para o tratamento da DP. Este estudo segue a lista de verificação das Diretrizes para o Relato de Estudos de Confiabilidade e Concordância (GRRAS).^8^ O estudo foi aprovado pelo comitê de ética local (CAAE: 95350718.6.0000.5511), e todos os pacientes concederam consentimento informado por escrito para participação no estudo.

### Procedimentos

Os pacientes visitaram o laboratório em três ocasiões distintas, no mesmo horário do dia, com um intervalo mínimo de 72 horas entre as visitas, conforme descrito anteriormente.^7^ Os mesmos procedimentos experimentais foram empregados em todas as ocasiões. Antes de cada visita, os pacientes foram instruídos a não realizarem exercícios por 48 horas antes das sessões experimentais, a consumirem uma refeição leve duas horas antes, a tomarem sua medicação para DP 30 minutos antes e a não consumir bebidas com cafeína nos dias das sessões experimentais.

Em cada visita, ao chegar ao laboratório, os pacientes eram colocados em repouso, em decúbito dorsal. Após 10 minutos, as avaliações eram realizadas na seguinte ordem: PA braquial, PA central, rigidez arterial e VFC. Os dados foram coletados e analisados pelo mesmo pesquisador não cego.

### Desfechos

#### Pressão arterial

A PA braquial foi medida pelo método auscultatório, usando um esfigmomanômetro de mercúrio. Três medições consecutivas eram realizadas, até que diferenças menores que 4 mmHg fossem alcançadas entre as medições, com intervalos de 1 minuto entre elas, no braço menos afetado pela DP, e com o tamanho do manguito apropriado para a circunferência do braço do paciente. A média dos três valores foi calculada para análise dos dados.

A PA central foi avaliada pela técnica de análise da onda de pulso, registrada na artéria radial do braço menos afetado pela DP, usando tonometria de aplanação (SphygmoCor Atcor Medical, Sydney, Austrália),^9^ e a pressão de pulso central foi avaliada pela diferença entre a PA sistólica central e a PA diastólica central.

#### Rigidez arterial

Os parâmetros de rigidez arterial foram obtidos usando o dispositivo SphygmoCor (Atcor Medical, Sidney, Austrália).^10^ Para a rigidez arterial, as formas de onda de pressão nos locais da artéria carótida e femoral, obtidas por meio de tonometria de aplanação, simultaneamente com o registro do eletrocardiograma, foram usadas para calcular a velocidade da onda de pulso. A análise da onda de pulso obtida na artéria radial incluiu o índice de aumento e o índice de aumento 75 (unidades normalizadas correspondentes a uma frequência cardíaca de 75 bpm).

#### Variabilidade da frequência cardíaca

A modulação autonômica cardíaca foi avaliada por meio da análise da VFC^11^ Os intervalos R-R foram registrados usando um monitor de frequência cardíaca (V800; Polar Electro, Kempele, Finlândia) e a análise dos dados foi realizada com software específico (Kubios HRV, Kubios Oy, Kuopil, Finlândia). Os seguintes índices foram obtidos: i) desvio padrão de todos os intervalos R-R (SDNN; marcador da VFC total); ii) raiz quadrada média das diferenças quadradas entre intervalos R-R normais adjacentes (RMSSD; marcador de modulação vagal predominante); iii) componente de baixa frequência da variabilidade do intervalo R-R (LF_R-R_; marcador de modulação simpática predominante); iv) componente de alta frequência da variabilidade do intervalo R-R (HF_R-R_; marcador de modulação vagal); e v) equilíbrio simpatovagal cardíaco (LF/HF). LF_R-R_ e HF_R-R_ foram expressos em unidades absolutas e normalizadas.

## Análises estatísticas

Os valores são expressos como média ± desvio padrão. A normalidade de todos os dados foi verificada pelo teste de Shapiro-Wilk, com dados não normais sendo transformados via logaritmo natural (ln) antes de análises posteriores. A presença de viés sistemático foi avaliada, comparando os valores entre as três sessões experimentais usando ANOVAs unidirecionais. Um teste post-hoc de Newman-Keuls foi empregado quando um efeito principal foi identificado. A confiabilidade foi examinada por meio do modelo misto bidirecional de correlação de coeficiente intraclasse (CCI),^12^ com resultados variando de 0,0 a 1,0 e valores mais altos indicando melhor confiabilidade. A concordância foi avaliada por gráficos de Bland-Altman, com limites de concordância (LC) de 95% e erro padrão de medição (EPM), expressos nas unidades reais de medição. Para melhorar a comparabilidade entre as diferentes variáveis estudadas, o EPM também foi expresso como uma proporção dos valores medidos (EPM%), calculados pela seguinte equação: EPM% = {EPM/[(valor médio da sessão 1 + valor médio da sessão 2 + valor médio da sessão 3)/3]}. Valores menores de EPM% indicam melhor concordância. A alteração mínima detectável foi calculada usando a seguinte equação: 1,64×√2×EPM.

## Resultados

As características da amostra são descritas na Tabela 1. Todos os pacientes eram homens e a maioria (n = 15; 75% dos pacientes) estava nos estágios 2 e 2,5 modificados de Hoehn & Yahr. Todos os pacientes estavam tomando levodopa e 40% estavam usando um agonista da dopamina.

**Tabela 1:** – Descrição das características da amostra (n=20)

Características	Valores
**Idade (anos)**	65±7
**IMC (kg/m^**2**^)**	28,0±4,4
**Características da DP**	
Duração da doença (anos)	6,5±3,5
***Hoehn & Yahr modificado***	
Estágios 1-1,5 – n (%)	3 (15)
Estágio 2-2,5 – n (%)	15 (75)
Estágio 3 – n (%)	2 (10)
**Tratamento farmacológico da DP**	
Levodopa/Carbidopa – n (%)	20 (100)
Agonista da Dopamina – n (%)	8 (40)
Amantadina – n (%)	5 (25)
Selegilina – n (%)	3 (15)
**Comorbidades**	
Hipertensão	4 (20)
Dislipidemia	2 (10)
Diabetes *mellitus*	0 (0)
**Uso de outros medicamentos**	
IECA	1 (5)
BRA	3 (15)
Diuréticos	1 (5)
Estatinas	2 (10)

Os valores são média ± DP ou número (porcentagem). DP: doença de Parkinson; IECA: inibidor da enzima conversora da angiotensina; BRA: bloqueador do receptor da angiotensina; IMC: índice de massa corporal.

### Pressão arterial

**Figura 1:**
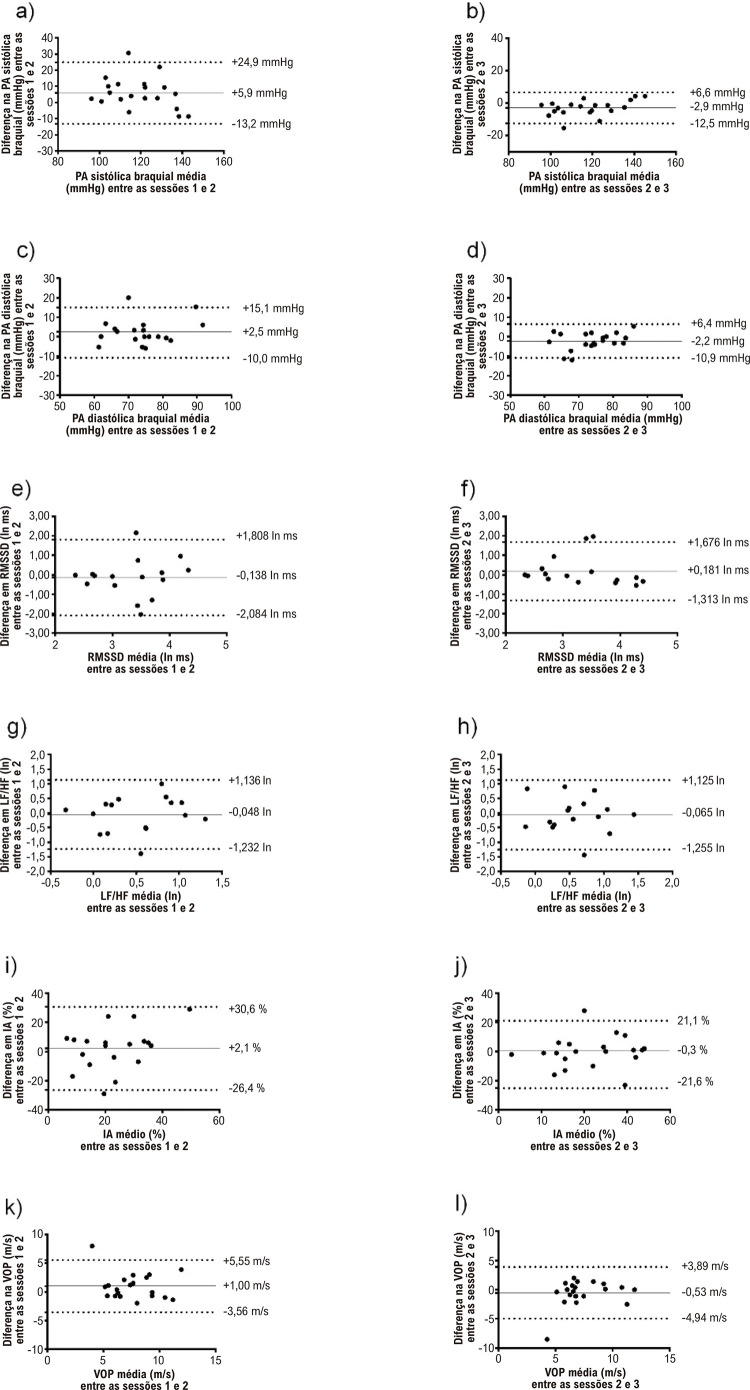
– Gráficos de Bland e Altman (viés sistemático ± limites de concordância) para valores individuais de pressão arterial sistólica braquial, pressão arterial diastólica braquial, raiz quadrada média de diferenças sucessivas entre batimentos cardíacos normais (RMSSD), balanço simpatovagal cardíaco (LF/HF), índice de aumento (IA) e velocidade da onda de pulso (VOP). ln: logaritmo natural.

Foi observado um efeito principal significativo da sessão (p<0,05) sobre a PA sistólica braquial e central. Para a PA sistólica braquial e central, a análise post-hoc revelou valores maiores na visita 1 quando comparada às visitas 2 e 3. Não houve diferenças significativas para a PA diastólica braquial e central (p>0,05) ou pressão de pulso central (p>0,05) entre as visitas experimentais. Os CCIs variaram de 0,725 (pressão de pulso central) a 0,921 (PA sistólica braquial) e EPM% de 2,5% (PA sistólica braquial) a 18,0% (pressão de pulso central) (Tabela 2). Os gráficos de Bland–Altman indicaram baixa concordância entre as visitas 1 e 2 e boa concordância entre as visitas 2 e 3 (Figura 1).

**Tabela 2:** – Reprodutibilidade das avaliações de pressão arterial, rigidez arterial e variabilidade da frequência cardíaca em pacientes com doença de Parkinson

Variável	Sessão 1	Sessão 2	Sessão 3	ANOVA unidirecional Valor-p	CCI (IC de 95%)	EPM (EPM%)	DMD
**Pressão arterial**							
PA sistólica braquial (mmHg)	122±13*	116±16	120±15	0,029	0,921 (0,834 a 0,966)	2,9 (2,5)	6,8
PA diastólica braquial (mmHg)	75±9	73±8	75±7	0,132	0,892 (0,772 a 0,954)	2,1 (2,8)	4,8
PA sistólica central (mmHg)	113±15*	106±15	109±14	0,039	0,842 (0,666 a 0,932)	5,5 (5,0)	12,6
PA diastólica central (mmHg)	75±8	73±9	76±8	0,068	0,887 (0,761 a 0,952)	2,2 (2,9)	5,0
Pressão de pulso central (mmHg)	37±11	33±11	33±11	0,218	0,725 (0,422 a 0,883)	6,2 (18,0)	14,3
**Rigidez arterial**							
IA (%)	23±16	21±11	22±10	0,686	0,781 (0,538 a 0,906)	5,6 (25,2)	13,0
IA75 (%)	21±12	20±9	18±8	0,433	0,787 (0,551 a 0,909)	4,5 (22,9)	10,4
VOP (m/s)	8,1±2,23	7,5±2,07	7,7±2,07	0,210	0,886 (0,754 a 0,952)	0,6 (7,3)	1,3
**Variabilidade da frequência cardíaca**							
Intervalo R-R (ms)	916±129	888±106	939±111	0,150	0,820 (0,584 a 0,932)	46,7 (5,1)	108,3
ln SDNN (ms)	3,04±0,73	3,20±0,75	3,04±0,74	0,597	0,795 (0,527 a 0,922)	0,32 (10,3)	0,74
ln RMSSD (ms)	3,28±0,81	3,42±0,73	3,24±0,85	0,683	0,677 (0,255 a 0,878)	0,50 (15,0)	1,15
ln LF_R-R_ (ms^2^)	4,82±1,30	4,93±1,38	5,13±1,43	0,648	0,765 (0,459 a 0,911)	0,66 (13,3)	1,53
ln HF_R-R_ (ms^2^)	4,75±1,39	4,25±1,24	4,52±1,26	0,191	0,854 (0,664 a 0,945)	0,43 (9,6)	1,00
LF_R-R_ (“nu”, %)	61±13	63±12	64±12	0,768	0,786 (0,506 a 0,919)	6,7 (10,8)	15,6
HF_R-R_ (“nu”, %)	39±13	37±12	36±12	0,755	0,568 (0,003 a 0,837)	9,6 (25,6)	22,2
*ln LF/HF_R-R_*	*0,50±0,58*	*0,54±0,51*	*0,61±0,55*	*0,779*	*0,583 (0,038 a 0,842)*	*0,42 (76,0)*	*0,97*

Os valores são média ± DP. IC 95%: intervalo de confiança de 95%; IA: índice de aumento; IA75: índice de aumento normalizado pela frequência cardíaca de 75 bpm; PA: pressão arterial; HF_R-R_: banda de alta frequência da variabilidade do intervalo R-R; CCI: coeficiente de correlação intraclasse; LF/HF_R-R_: razão entre as bandas de baixa e alta frequência; LF_R-R_: banda de baixa frequência da variabilidade do intervalo R-R; ln: logaritmo natural; DMD: diferença mínima detectável; VOP: velocidade da onda de pulso; RMSSD: raiz quadrada média das diferenças quadradas entre intervalos R-R normais adjacentes; SDNN: desvio padrão de todos os intervalos R-R; EPM: erro padrão de medição; EPM%: erro padrão de medição normalizado pelos valores médios das medições. *: significativamente diferente das Sessões 2 e 3 (p<0,05).

### Rigidez arterial

Não houve diferenças significativas entre as visitas experimentais nos valores médios de qualquer variável de rigidez arterial (p>0,05). Os CCIs variaram de 0,781 (índice de aumento) a 0,886 (velocidade da onda de pulso) e EPM% de 7,3% (velocidade da onda de pulso) a 25,2% (índice de aumento) (Tabela 2). Os gráficos de Bland-Altman indicaram concordância moderada a boa entre as visitas para os parâmetros de índice de aumento e velocidade da onda de pulso, respectivamente (Figura 1).

### Variabilidade da frequência cardíaca

Não houve diferenças significativas entre as visitas experimentais nos valores médios de qualquer variável (p>0,05). Os CCIs variaram de 0,568 (HF_R-R_ normalizado) a 0,854 (ln HF_R-R_ em unidades absolutas) e EPM% de 5,1% (intervalo R-R) a 76,0% (LF/HF_R-R_) (Tabela 2). Os gráficos de Bland–Altman indicaram concordância moderada entre as visitas para a maioria dos parâmetros de VFC, exceto para LF/HF_R-R_ (Figura 1).

## Discussão

Os principais resultados do estudo atual foram que, em pacientes com DP, a PA apresentou baixa confiabilidade intra-avaliador e concordância entre as visitas 1 e 2 e boa confiabilidade e concordância entre as visitas 2 e 3. Em geral, a confiabilidade intra-avaliador e a concordância dos índices de rigidez arterial e VFC foram aceitáveis.

As medidas de PA braquial e central apresentaram baixa confiabilidade intra-avaliador e concordância entre as visitas 1 e 2. A PA sistólica central e braquial estavam elevadas na primeira visita experimental, indicando a presença de viés sistemático. As diretrizes clínicas^13,14^ recomendam a medição da PA em duas ou mais visitas para determinação da PA em repouso, a fim de minimizar fatores intervenientes, como o ambiente do consultório, procedimentos de medição e outros. Essa recomendação está alinhada com nossos dados realizados em pacientes com DP, que demonstraram reduções na PA da primeira para a segunda visita e estabilização nessa variável entre a segunda e a terceira visita. Esse resultado indica que os estudos com pacientes com DP devem empregar uma sessão de familiarização antes das sessões experimentais para obtenção de dados estáveis de PA sistólica.

Anteriormente, foram relatadas boa confiabilidade e concordância para PA braquial^15^ e central em indivíduos saudáveis.^16^ Assim, os resultados atuais sugerem que, embora a DP esteja associada a uma reatividade comprometida da PA (por exemplo, hipotensão ortostática e hipertensão supina),^1,17^ isso não se traduz em baixa confiabilidade e concordância nas avaliações de PA em repouso após a segunda visita.

Foram observadas confiabilidade e concordância de moderadas a boa para avaliações de rigidez arterial, com a velocidade da onda de pulso sendo a medida mais confiável. Esses resultados estão de acordo com dados anteriores, obtidos em um estudo com idosos saudáveis.^18^ Esse resultado é relevante, pois a VOP tem sido considerada um marcador de risco cardiovascular.^19,20^ Portanto, os dados atuais indicam que a DP não prejudica a confiabilidade e a concordância intra-avaliador para rigidez arterial. A capacidade da DP em afetar diretamente os resultados de rigidez arterial continua sendo um tópico controverso.^21,22^

Os índices de VFC apresentaram confiabilidade e concordância intra-avaliador aceitáveis, exceto para ln LF/HF_R-R_. De fato, a magnitude do EPM foi semelhante entre as variáveis transformadas em log; no entanto, quando o EPM foi considerado em relação aos respectivos valores médios, ln LF/HF_R-R_ exibiu um EPM% muito grande. Além disso, recomenda-se cautela ao empregar o HF_R-R_ normalizado para rastrear alterações de VFC em pacientes com DP. No entanto, os resultados atuais são semelhantes àqueles obtidos por estudos anteriores^23-27^ em indivíduos sem DP, o que sugere que a DP não afeta a confiabilidade e a concordância da VFC. Assim, embora os pacientes com DP apresentem valores anormais em alguns índices de VFC,^28^ os resultados atuais sugerem que a variabilidade “entre dias” desses parâmetros não parece ser aumentada em relação a indivíduos sem DP.

É importante discutir nossos resultados de DMD à luz dos tamanhos de efeito observados em estudos clínicos anteriores com pacientes com DP. Resultados mistos foram relatados em relação à responsividade da PA a intervenções clínicas, com resultados positivos^29^ ou nenhum efeito.^30-33^ Di Francisco-Donoghue et al. (2019)^29^ observaram um aumento na PA sistólica de 38 mmHg, 60 minutos após uma intervenção com goma de nicotina, em pacientes com DP que sofrem com hipotensão arterial. Outro estudo^34^ avaliou os efeitos cardiovasculares agudos da administração oral de levodopa em pacientes com DP moderada e relatou uma diminuição na PA sistólica de 19 mmHg. Este efeito é maior do que a DMD determinada no estudo atual para PA sistólica (6,8 mmHg), indicando que mudanças individuais verdadeiras podem ser detectadas em pacientes com DP. Poucos estudos clínicos também empregaram índices de VFC como desfechos, com relatos de resultados positivos^17^ ou nenhum efeito.^31,35^ O único estudo que encontrou resultados positivos estatisticamente significativos avaliou os efeitos do treinamento de resistência progressivo e observou mudanças de 14 “nu” na LF _R-R_ (“nu”).^17^ Este efeito é ligeiramente menor do que a DMD (16 “nu”) observada no estudo atual, sugerindo que verdadeiras mudanças na LF _R-R_ (“nu”) podem ser detectadas em um número razoável de indivíduos, já que muitos pacientes no estudo de Kanegusuku et al. (2017)^17^ mostraram alterações ≥ 16 “nu”. Em contraste, existe uma escassez de estudos investigando a resposta da rigidez arterial às intervenções clínicas. Com base nos resultados atuais da DMD, estudos futuros com pacientes com DP devem analisar as respostas individuais, examinando quais pacientes apresentaram mudanças reais após as intervenções (mudanças observadas ≥ DMD), principalmente nos parâmetros de PA e VFC. Estudos ainda são necessários sobre a resposta da rigidez arterial às intervenções clínicas.

Os resultados atuais têm implicações significativas para pesquisas e cenários clínicos. Os dados de EPM podem ser utilizados para calcular os tamanhos de amostra necessários para estudos clínicos,^36^ enquanto a DMD representa a menor quantidade de mudança necessária entre testes repetidos para indicar uma mudança real ao avaliar respostas individuais a intervenções.^37^ Sendo assim, os valores de EPM e DMD determinados no estudo atual devem ser considerados ao conduzir pesquisas ou avaliações clínicas em pacientes com DP. Além disso, recomenda-se que pacientes com DP realizem uma sessão de familiarização da medição da PA para garantir a obtenção de dados estáveis de PA sistólica.

Por fim, é importante mencionar as limitações do estudo atual. Alguma cautela é necessária antes de extrapolar os resultados atuais para pacientes com DP com outras características, como mulheres, pacientes em estágios mais graves de DP e aqueles com comorbidades cardiovasculares. Outra limitação do estudo atual inclui a falta de um grupo de controle comparativo, composto por uma amostra pareada de indivíduos sem DP. Por outro lado, é importante destacar que o estudo atual empregou uma análise abrangente de reprodutibilidade, envolvendo avaliações sistemáticas de viés, confiabilidade e concordância, bem como três sessões experimentais.

## Conclusão

Em pacientes com DP, as avaliações da PA central e braquial mostraram baixa confiabilidade e concordância intra-avaliador entre as visitas 1 e 2 e boa confiabilidade e concordância entre as visitas 2 e 3. As avaliações de rigidez arterial apresentaram confiabilidade e concordância intra-avaliador aceitáveis, com a velocidade da onda de pulso sendo o índice mais confiável. Os índices de VFC apresentaram confiabilidade e concordância intra-avaliador aceitáveis, exceto pela relação LF/HF_R-R_.
