# Supplementary material for: Confiabilidade e Concordância Intra-Avaliador das Avaliações da Pressão Arterial, Rigidez Arterial e Variabilidade da Frequência Cardíaca em Pacientes com Doença de Parkinson
Source: Arq Bras Cardiol. 2024 Oct 23;121(10):e20240132. [Article in Portuguese] doi: 10.36660/abc.20240132 (PMC11634208; doi:10.36660/abc.20240132)
Supplement: Translation

# Translation: Intrarater Reliability and Agreement of Blood Pressure, Arterial Stiffness, and Heart Rate Variability Assessments in Patients With Parkinson’s Disease

**DOI:** 10.36660/abc.20240132i

**Authors:** Verônica de Fátima Souza Lima, Rafael Yokoyama Fecchio, Maria Elisa Pimentel Piemonte, Marilia de Almeida Correia, Hélcio Kanegusuku, Raphael Mendes Ritti-Dias

**Affiliations:** 1 Brasil Universidade Nove de Julho, São Paulo, SP – Brasil; 2 Brasil Universidade de São Paulo, São Paulo, SP – Brasil; 3 Brasil Hospital Israelita Albert Einstein, São Paulo, SP – Brasil

**Keywords:** Parkinson Disease, Blood Pressure, Vascular Stiffness, Heart Rate

## Abstract

To assess the intrarater reliability and agreement of blood pressure (BP), arterial stiffness, and heart rate variability (HRV) assessments in patients with Parkinson`s disease (PD). Twenty patients with PD visited the laboratory three times, during which brachial and central BP (auscultatory and applanation tonometry, respectively), arterial stiffness (carotid-femoral pulse wave velocity and augmentation index), and HRV assessments were performed at rest. Brachial and central systolic BP presented greater values on visit 1 when compared to visits 2 and 3 (122±13 vs. 116±16 vs. 120±15, p=0.029). There were no significant differences (p>0.05) among the experimental visits for other parameters. Brachial and central BP showed an intraclass correlation coefficient (ICC) above 0.842 and a standard error of measurement (SEM) lower than 5.0%. Bland–Altman plots indicated low agreement between visits 1 and 2 and good agreement between visits 2 and 3. Arterial stiffness indices exhibited ICC values between 0.781 and 0.886, and SEM ranged from 7.3% to 25.2%. Bland–Altman plots indicated moderate to good agreement among visits for arterial stiffness parameters. HRV indices presented ICC values ranging from 0.558 to 0.854 and SEM values ranging from 5.1% to 76.0%. Bland–Altman plots indicated moderate agreement among visits for HRV parameters. In PD patients, brachial and central BP present low intrarater reliability and agreement between visits 1 and 2 and good intrarater reliability and agreement between visits 2 and 3. In general, arterial stiffness and HRV assessments present acceptable intrarater reliability and agreement among visits, except for cardiac sympathovagal balance.

## Introduction

Parkinson’s Disease (PD) is a progressive neurodegenerative disorder, characterized by dysfunction of the nigrostriatal dopaminergic system, resulting in motor symptoms such as bradykinesia, resting tremor, rigidity, and postural instability,^1^ which reduce the quality of life of these patients.^2^ PD also involves degeneration of the peripheral autonomic nervous system, including decreases in noradrenergic fibers and norepinephrine availability in the myocardium, which contributes to cardiovascular dysregulation.^1^ Emerging evidence has shown that alterations in cardiovascular function and regulation might be associated with the debilitating symptoms of PD.

In PD patients, elevated resting blood pressure (BP) increases the risk of mild cognitive impairment fourfold.^3^ Increased arterial stiffness is associated with orthostatic hypotension and supine hypertension,^4^ while reduced cardiac autonomic modulation is linked to freezing of gait^5^ and cognitive impairment.^6^ Consequently, there is growing interest in evaluating cardiovascular outcomes in PD patients. Despite this, it is unclear whether assessments of cardiovascular variables present good intrarater reliability and agreement, which are essential factors for assessing true changes in response to interventions. Therefore, in the current study, we evaluated the intrarater reliability and agreement of brachial and central BP (auscultatory and applanation tonometry, respectively), arterial stiffness carotid-femoral pulse wave velocity and augmentation index, and heart rate variability (HRV) assessments in patients with PD.

## Methods

### Participants

The present study is a secondary analysis of data obtained from a previously published study.^7^ For that study, the power obtained was 0.99%, with an effect size of 0.70 for brachial systolic BP response for the three experimental sessions performed by 20 patients, considering an alpha error of 0.05 (G*Power v. 3.1.9.4, Universität Kiel, Germany). Therefore, the sample size of the present study was the same as the previous one. Non-probability sampling was used to recruit patients from the Brazil Parkinson Association in São Paulo, Brazil. The eligibility criteria included having a confirmed diagnosis of PD, being at least 50 years old, not being in PD stages 4-5 (according to the modified Hoehn and Yahr scale), not having any other neurological disorder apart from PD, not having any cardiac disease or electrocardiographic abnormalities at rest and during maximal exercise tests, and not using medications that directly impact cardiac autonomic regulation (e.g., beta-blockers), except for those prescribed for PD treatment. This study follows the Guidelines for Reporting Reliability and Agreement Studies (GRRAS) checklist.^8^ The study was approved by the local ethics committee (CAAE: 95350718.6.0000.5511), and written informed consent was obtained from all patients.

### Procedures

Patients visited the laboratory on three separate occasions, at the same time of day, with an interval of at least 72 hours between visits, as previously described.^7^ The same experimental procedures were employed on all occasions. Prior to each visit, patients were instructed not to perform exercise for 48 hours before experimental sessions, to consume a light meal two hours before, to take their PD medication 30 minutes before, and to refrain from consuming caffeinated drinks on experimental session days.

At each visit, when arriving at the laboratory, patients were placed at rest in the supine position. After 10 minutes, the assessments were performed in the following order: brachial BP, central BP, arterial stiffness, and HRV. Data were collected and analyzed by the same experienced researcher, who was not blinded.

### Outcomes

#### Blood Pressure

Brachial BP was measured by the auscultatory method using a mercury sphygmomanometer. Three consecutive measurements were performed until differences of less than 4 mmHg were reached among the measurements, with 1-minute intervals between them on the arm less affected by PD and with the appropriate cuff size for the arm circumference. The average of the three values was calculated for data analysis.

Central blood pressure was assessed by the pulse wave analysis technique, recorded in the radial artery of the arm less affected by PD, using applanation tonometry (SphygmoCor Atcor Medical, Sydney, Australia),^9^ and the difference between central systolic BP and central diastolic BP assessed the central pulse pressure.

#### Arterial Stiffness

Arterial stiffness parameters were obtained using the SphygmoCor device (Atcor Medical, Sidney, Australia).^10^ For arterial stiffness the pressure waveforms at the carotid and femoral artery sites obtained using applanation tonometry simultaneously with electrocardiogram recording were used to calculate pulse wave velocity. The pulse wave analysis obtained in the radial artery included the augmentation index and augmentation index 75 (normalized units corresponding to a heart rate of 75 bpm).

#### Heart rate variability

Cardiac autonomic modulation was evaluated through the analysis of HRV.^11^ R-R intervals were registered using a heart rate monitor (V800; Polar Electro, Kempele, Finland), and data analysis was performed with specific software (Kubios HRV, Kubios Oy, Kuopil, Finland). The following indices were obtained: i) standard deviation of all R-R intervals (SDNN; marker of total HRV); ii) root mean square of the squared differences between adjacent normal R-R intervals (RMSSD; marker of predominant vagal modulation); iii) low-frequency component of R-R interval variability (LF_R-R_; marker of predominant sympathetic modulation); iv) high-frequency component of R-R interval variability (HF_R-R_; marker of vagal modulation); and v) cardiac sympathovagal balance (LF/HF). LF_R-R_ and HF_R-R_ were expressed in both absolute and normalized units.

## Statistical analyses

Values are expressed as mean ± standard deviation. The normality of all data was checked by the Shapiro-Wilk test, with non-normal data being transformed via natural logarithm (ln) prior to further analysis. The presence of systematic bias was evaluated, comparing the values between the three experimental sessions using one-way ANOVAs. A Newman-Keuls post-hoc test was employed when a main effect was identified. Reliability was examined via the intraclass coefficient correlation (ICC) two-way mixed model,^12^ with results ranging from 0.0 to 1.0 and higher values indicating better reliability. The agreement was evaluated by Bland-Altman plots, with 95% limits of agreement (LOA) and the standard error of measurement (SEM) expressed in the actual units of measurement. To improve comparability between the different variables studied, SEM was also expressed as a proportion of the measured values (SEM%), calculated using the following equation: SEM% = {SEM / [(session 1 mean value + session 2 mean value + session 3 mean value) / 3]}. Lower SEM% values indicate better agreement. The minimal detectable change was calculated using the following equation: 1.64×√2×SEM.

## Results

The sample characteristics are described in Table 1. All patients were men, and the majority (n=15; 75% of patients) were in Hoehn & Yahr modified stages 2 and 2.5. All patients were taking levodopa, and 40% were using a dopamine agonist.

**Table 1:** – Sample characteristics description (n=20)

Characteristics	Values
**Age (years old)**	65±7
**BMI (kg/m^**2**^)**	28.0±4.4
**PD characteristics**	
Disease duration (years)	6.5±3.5
***Hoehn & Yahr modified***	
Stages 1-1.5 – n (%)	3 (15)
Stage 2-2.5 – n (%)	15 (75)
Stage 3 – n (%)	2 (10)
**PD pharmacological treatment**	
Levodopa/Carbidopa – n (%)	20 (100)
Dopamine Agonist – n (%)	8 (40)
Amantadine – n (%)	5 (25)
Selegiline – n (%)	3 (15)
**Comorbidities**	
Hypertension	4 (20)
Dyslipidemia	2 (10)
Diabetes Mellitus	0 (0)
**Use of other medications**	
ACEi	1 (5)
ARB	3 (15)
Diuretics	1 (5)
Statins	2 (10)

Values are mean ± SD or number (percentage). PD: Parkinson`s disease; ACEi: angiotensin-converting enzyme inhibitor; ARB: angiotensin receptor blocker; BMI: body mass index.

### Blood pressure

There was a significant main effect of the session (p<0.05) for brachial and central systolic BP. For both brachial and central systolic BP, post-hoc analysis revealed greater values in visit 1 when compared to visits 2 and 3. There were no significant differences for brachial and central diastolic BP (p>0.05) or central pulse pressure (p>0.05) among the experimental visits. ICCs ranged from 0.725 (central pulse pressure) to 0.921 (brachial systolic BP), and SEM% from 2.5% (brachial systolic BP) to 18.0% (central pulse pressure) (Table 2). Bland–Altman plots indicated low agreement between visits 1 and 2 and good agreement between visits 2 and 3 (Figure 1).

**Table 2:** – Reproducibility of blood pressure, arterial stiffness, and heart rate variability assessments in Parkinson`s Disease patients

Variable	Session 1	Session 2	Session 3	One-way ANOVA p-value	ICC (95% CI)	SEM (SEM%)	MDD
**Blood pressure**							
Brachial systolic BP (mmHg)	122±13*	116±16	120±15	0.029	0.921 (0.834 to 0.966)	2.9 (2.5)	6.8
Brachial diastolic BP (mmHg)	75±9	73±8	75±7	0.132	0.892 (0.772 to 0.954)	2.1 (2.8)	4.8
Central systolic BP (mmHg)	113±15*	106±15	109±14	0.039	0.842 (0.666 to 0.932)	5.5 (5.0)	12.6
Central diastolic BP (mmHg)	75±8	73±9	76±8	0.068	0.887 (0.761 to 0.952)	2.2 (2.9)	5.0
Central pulse pressure (mmHg)	37±11	33±11	33±11	0.218	0.725 (0.422 to 0.883)	6.2 (18.0)	14.3
**Arterial stiffness**							
AIx (%)	23±16	21±11	22±10	0.686	0.781 (0.538 to 0.906)	5.6 (25.2)	13.0
AIx75 (%)	21±12	20±9	18±8	0.433	0.787 (0.551 to 0.909)	4.5 (22.9)	10.4
PWV (m/s)	8.1±2.23	7.5±2.07	7.7±2.07	0.210	0.886 (0.754 to 0.952)	0.6 (7.3)	1.3
**Heart rate variability**							
R-R interval (ms)	916±129	888±106	939±111	0.150	0.820 (0.584 to 0.932)	46.7 (5.1)	108.3
ln SDNN (ms)	3.04±0.73	3.20±0.75	3.04±0.74	0.597	0.795 (0.527 to 0.922)	0.32 (10.3)	0.74
ln RMSSD (ms)	3.28±0.81	3.42±0.73	3.24±0.85	0.683	0.677 (0.255 to 0.878)	0.50 (15.0)	1.15
ln LF_R-R_ (ms^2^)	4.82±1.30	4.93±1.38	5.13±1.43	0.648	0.765 (0.459 to 0.911)	0.66 (13.3)	1.53
ln HF_R-R_ (ms^2^)	4.75±1.39	4.25±1.24	4.52±1.26	0.191	0.854 (0.664 to 0.945)	0.43 (9.6)	1.00
LF_R-R_ (nu, %)	61±13	63±12	64±12	0.768	0.786 (0.506 to 0.919)	6.7 (10.8)	15.6
HF_R-R_ (nu, %)	39±13	37±12	36±12	0.755	0.568 (0.003 to 0.837)	9.6 (25.6)	22.2
ln LF/HF_R-R_	0.50±0.58	0.54±0.51	0.61±0.55	0.779	0.583 (0.038 to 0.842)	0.42 (76.0)	0.97

Values are mean ± SD. 95% CI: 95% confidence interval; AIx: Augmentation index; AIx75: augmentation index normalized by 75 bpm heart rate; BP: blood pressure; HF_R-R_: high frequency band of R-R interval variability; ICC: intraclass correlation coefficient; LF/HF_R-R_: ratio between the low and the high frequency bands; LF_R-R_: low frequency band of R-R interval variability; ln: natural logarithm; MDD: minimal detectable difference; PWV: pulse wave velocity; RMSSD: root mean square of the squared differences between adjacent normal R-R intervals; SDNN: standard deviation of all R-R intervals; SEM: standard error of measurement; SEM%: standard error of measurement normalized by measurement mean values. *: significantly different from Sessions 2 and 3 (p<0.05).

**Figure 1:** – Bland and Altman plots (systematic bias ± limits of agreement) for individual values of brachial systolic blood pressure, brachial diastolic blood pressure, root mean square of successive differences between normal heartbeats (RMSSD), cardiac sympathovagal balance (LF/HF), augmentation index (AIx) and pulse wave velocity (PWV). Ln: natural logarithm.

### Arterial stiffness

There were no significant differences among experimental visits in the mean values of any arterial stiffness variable (p>0.05). ICCs ranged from 0.781 (augmentation index) to 0.886 (pulse wave velocity), and SEM% from 7.3% (pulse wave velocity) to 25.2% (augmentation index) (Table 2). Bland–Altman plots indicated moderate to good agreement among visits for the augmentation index and pulse wave velocity parameters, respectively (Figure 1).

### Heart rate variability

There were no significant differences among experimental visits in the mean values of any variable (p>0.05). The ICCs ranged from 0.568 (normalized HF_R-R_) to 0.854 (ln HF_R-R_ in absolute units) and SEM% from 5.1% (R-R interval) to 76.0% (LF/HF_R-R_) (Table 2). Bland–Altman plots indicated moderate agreement among visits for the majority of HRV parameters, except for LF/HF_R-R_ (Figure 1).

## Discussion

The main findings of the current study were that, in PD patients, BP presented low intrarater reliability and agreement between visits 1 and 2 and good reliability and agreement between visits 2 and 3. In general, the intrarater reliability and agreement of arterial stiffness and HRV indices were acceptable.

Brachial and central BP measurements presented low intrarater reliability and agreement between visits 1 and 2. Both central and brachial systolic BP were elevated in the first experimental visit, indicating the presence of systematic bias. Clinical guidelines^13,14^ recommend the measurement of BP in two or more visits to determine resting BP in order to minimize intervenient factors, such as the office environment, BP measurement procedures, and others. This is aligned with our data performed in PD patients, which demonstrated decreases in BP from the first to the second visit and stabilization in this variable between the second and third visits. This result indicates that trials with PD patients should employ one familiarization session before experimental sessions in order to obtain stable systolic BP data.

Good reliability and agreement were previously reported for brachial^15^ and central BPs in healthy individuals.^16^ Thus, the current results suggest that although PD is associated with impaired BP reactivity (e.g., orthostatic hypotension and supine hypertension),^1,17^ this does not translate into low reliability and agreement of resting BP assessments after the second visit.

Moderate to good reliability and agreement were found for arterial stiffness assessments, with pulse wave velocity being the most reliable measurement. These results are in line with previous data obtained in healthy older adults.^18^ This result is relevant since pulse wave velocity has been considered a marker of cardiovascular risk.^19,20^ Thus, the present data indicate that PD does not impair intrarater reliability and agreement for arterial stiffness. Indeed, it remains controversial whether PD directly affects arterial stiffness outcomes.^21,22^

The HRV indices presented acceptable intrarater reliability and agreement, except for ln LF/HF_R-R_. Indeed, the SEM magnitude was similar across log-transformed variables; however, when SEM was considered in relation to the respective mean values, ln LF/HF_R-R_ exhibited a very large SEM%. Additionally, some caution is also required when employing normalized HF_R-R_ for tracking HRV changes in PD patients. Nevertheless, the present results are similar to those obtained by previous studies^23-27^ in individuals without PD, which suggests that PD does not affect HRV reliability and agreement. Thus, although PD patients present abnormal values in some HRV indices,^28^ the current results suggest that the between-day variability of these parameters does not seem to be augmented in relation to non-PD individuals.

It is important to discuss our MDD results in light of the effect sizes found in previous clinical trials with PD patients. Mixed results have been reported regarding the responsiveness of BP to clinical interventions, with positive results^29^ or no effects.^30-33^ DiFrancisco-Donoghue et al.(2019)^29^ found an increase in systolic BP of 38 mmHg 60 minutes after a nicotine gum intervention in PD patients who suffer from low BP. Another study^34^ assessed the acute cardiovascular effects of levodopa oral administration in moderate PD patients and reported a decrease in systolic BP of 19 mmHg. This effect is greater than the MDD determined in the current study for systolic BP (6.8 mmHg), indicating that true individual changes can be detected in PD patients. Few clinical trials have also employed HRV indices as outcomes, with reports of both positive results^17^ or no effects.^31,35^ The only study that found statistically significant positive results evaluated the effects of progressive resistance training and found changes of 14 nu in LF _R-R_ (nu).^17^ This effect is slightly lower than the MDD (16 nu) found in the current study, suggesting that true changes in LF _R-R_ (nu) can be detected in a reasonable number of individuals, as many patients in the study by Kanegusuku et al. (2017)^17^ showed changes ≥ 16 nu. In contrast, there is a lack of studies investigating arterial stiffness responsiveness to clinical interventions. Based on the current MDD results, future studies with PD patients should analyze the individual responses, examining which patients showed true changes after interventions (observed changes ≥ MDD), mainly in BP and HRV parameters. Studies are still needed regarding arterial stiffness responsiveness to clinical interventions.

The current results have significant implications for both research and clinical settings. SEM data can be utilized for calculating the sample sizes required for clinical trials,^36^ while MDD represents the smallest amount of change required between repeated tests to indicate a real change when evaluating individual responses to interventions.^37^ Therefore, the SEM and MDD values determined in the current study should be considered when conducting research or clinical evaluations in PD patients. Furthermore, it is recommended that PD patients perform one familiarization session on the measurement of BP in order to obtain stable systolic BP data.

Lastly, it is important to mention the limitations of the current study. Some caution is required before extrapolating the current findings to PD patients with other characteristics, such as women, patients in more severe PD stages, and those with cardiovascular comorbidities. Another limitation of the current study includes the lack of a comparative control group composed of a paired sample of non-PD individuals. On the other hand, it is important to highlight that the current study employed a comprehensive reproducibility analysis involving systematic bias, reliability, and agreement evaluations, as well as three experimental sessions.

## Conclusion

In patients with PD, central and brachial BP assessments showed low intrarater reliability and agreement between visits 1 and 2 and good reliability and agreement between visits 2 and 3. Arterial stiffness assessments presented acceptable intrarater reliability and agreement, with pulse wave velocity being the most reliable index. HRV indices showed acceptable intrarater reliability and agreement, except LF/HF_R-R_.
